# Editorial: Canine and feline reproduction

**DOI:** 10.3389/fvets.2023.1120320

**Published:** 2023-02-09

**Authors:** Cristina Gobello

**Affiliations:** Faculty of Veterinary Sciences, Center of Reproductive Physiology, National University of La Plata, Argentina and National Scientific and Technical Research Council, La Plata, Argentina

**Keywords:** dog, cat, theriogenology, carnivore, wild

Felidae and Canidae are two families of the order Carnivora with both domestic and wild representatives. Felidae has been reproductively classified as seasonal polyestrous with induced ovulation. According to the IUCN Redlist of Threatened Species, ~75% of wild felids are listed as threatened or endangered. Canidae reproductive physiology is unique, for example, preovulatory luteinization of follicles, delayed oocyte maturation after ovulation, uterine-independent prolonged luteolysis, and long obligate anestrus between cycles, compared to other mammals. This has caused dog reproductive management to lag behind that of other species. Domestic dogs and cats have served as valuable models for developing reproductive technologies for managing rare, endangered carnivores.

Until the eighties, in most veterinary colleges, animal reproduction was mainly focused on farm animals. Although over the last 25 years there was an increase in canine and feline reproductive studies, further knowledge about physiology, pathology, diagnostic techniques, and therapeutics is still necessary to improve both breeding and contraception in these species.

The goal of this Research Topic is to publish high-quality peer-reviewed articles which update basic and applied knowledge on reproduction in domestic and wild carnivores. In this issue, there are 11 papers including three reviews, two clinical trials, and four questionnaire studies covering some of these topics.

The use of additives or micronutrients represents a feasible approach to improve sperm quality. There seems to be evidence linking gut microbiota and fertility. Mahiddine et al. test the effect of the oral administration of three commensal Lactobacillus spp. on canine sperm quality. They find that total and progressive motility, acrosome integrity, and other kinematic parameters were enhanced after the commensal lactobacilli administration.

Similarly, Aiudi et al. test the antioxidative activity of a mix of polyphenolic substances derived from the hydroxylation of Pinus Taeda lignin (PTHL) on canine blood and semen. PTHL improved the antioxidant status of animals as well as semen volume, concentration, and motility. These studies contain results useful for dog owners and breeders as well as for pet food producers in order to include substances with a potential beneficial effect on health and semen quality.

Prostatic diseases are very common in male dogs, accounting for up to 10% of cases submitted to veterinary practitioners. Palmieri et al. present a comprehensive and updated review describing the gross, cytological, and histological features of prostatic hyperplasia, prostatitis, prostatic cysts, and prostatic carcinoma in both canine and feline species.

Canine corpus luteum (CL) function is quite different from that of most other species. It has been hypothesized that estradiol, as well as its target genes, regulate canine CL lifespan, from formation through maintenance until regression. Pereira Bonfim Neto et al. develop an approach to uncover the relationship between 17b-estradiol and ESR1/ESR2 ratio in the regulation of canine CL throughout diestrus. ESR1 targets were greater at the beginning of diestrus, while the abundance of ESR2 targets was greater in the end. ESR1/ESR2 ratio shifted from an increasing to a decreasing pattern during the second half of the luteal phase. ERa-mediated positively regulated CL function at the beginning of diestrus and ERb-mediated effect contributed to luteal regression.

Vaginal cytology is a routinely used diagnostic tool in canine gynecological examinations which is useful for estrus cycle staging and the diagnosis of diseases. Vaginal cells vary under the influence of systemic estrogens. Attributing some subjectivity to this method, Reckers et al. standardize the identification of the different vaginal cell types through a tutorial flowchart. The use of this chart will lead to a high agreement among practitioners.

An accurate prediction of parturition day is of high importance to minimize neonatal death when planning labor assistance or cesarean sections (C-sections). However, the prediction of parturition day is still challenging when a pregnant bitch presents in the last week of gestation and the ovulation day is unknown. Although fetal gastrointestinal motility (FGM) has been shown to be useful to assess fetal maturity, there are still many aspects that should be unveiled. Siena et al. quantify FGM in relation to days before parturition (dbp), maternal size, and the sex ratio of pups. FGM increased in the last 5 dbp and it also was higher in small bitches. Conversely, parity and sex ratio did not seem to have an effect on FGM.

Dystocia in dogs is a frequent problem and C-sections, both as an emergency or planned, are a routine practice in small animals. Schrank et al. evaluate, through questionnaires, the incidence of C-sections and contributing factors comparing elective vs. emergency C-sections. These authors find that bitches with either small litters or which had prior C-sections have an increased risk. Primiparous bitches of advanced age and stillbirths presented higher emergency C-sections. A less popular breed, the Norwich Terrier, had an incidence of C-sections of more than 50%.

Conze et al., using the same methodology, compare fertility after C-section and compare it with natural parturition in dogs. They show that more than 90 % of the bitches became pregnant at the *first* breeding attempt either after C-sections or natural parturition. Bitches which underwent C-sections were more likely to have this intervention and more than 50% of bulldogs require C-sections at their *first* parturition.

The early experiences and environment during an animal's development can influence the appearance of specific diseases in adulthood. Factors such as nutrition, stress, or exposure to different microbes during critical windows of development can predispose the animal to certain diseases. An understanding of these factors is needed to guarantee health throughout life. In the review by Gaillard et al., the authors identify and discuss these factors with respect to adult obesity, chronic enteropathy, and behavioral disorders in dogs and cats, and areas of future research are suggested.

The popularity of brachycephalic dogs has been increasing in recent years. The extreme homozygosity of these breeds has led to an increase in the manifestation of deleterious genes that may lead to congenital malformations. There has been no data on the incidence of malformations in brachycephalic dogs compared with other breeds. Estevan et al. compare the frequency of malformations in brachycephalic dogs vs. other breeds. Overall malformations had an incidence of 6.77%, of which 87.5% were represented by brachycephalic breeds. The most common malformations in these breeds were cleft palate and anasarca.

Canids occupy the top of the food chain and are fundamental for the wild environmental balance in South America. On this continent, there are 11 species of canids and although some of them are threatened, little is known about their reproductive biology. Candido Carvalho et al. compile the current knowledge of South American wild canid estrous cycles, pregnancy, and parturition, as well as sexual behavior and ejaculate characteristics, identifying gaps in the knowledge which should be studied to facilitate the development of conservation programs.

Concluding, the results of the above-mentioned studies and reviews of this issue represent an enormous amount of new relevant data on andrological, gynecological, obstetrical, developmental, and genetic topics in both domestic and wild carnivores.

## Author contributions

The author confirms being the sole contributor of this work and has approved it for publication.

